# Ethnic disparities in multi-morbidity in women of reproductive age in the UK: a data linkage study

**DOI:** 10.1192/j.eurpsy.2022.1530

**Published:** 2022-09-01

**Authors:** R. Catalao, M. Ashworth, S. Hatch, L. Howard

**Affiliations:** 1 Institute of Psychiatry, Psychology and Neuroscience, Psychological Medicine, London, United Kingdom; 2 King’s College London, Primary Care Department, London, United Kingdom; 3 Institute of Psychiatry, Psychology and Neuroscience, Section Of Women’s Mental Health, London, United Kingdom

**Keywords:** Race, multi-morbidity, preconception, ethnic inequalities

## Abstract

**Introduction:**

Few studies have explored ethnic inequalities in physical and mental health in women at preconception.

**Objectives:**

Explore inequalities in multimorbidity in women of reproductive age.

**Methods:**

Data from Lambeth DataNet, anonymized primary care records of this ethnically diverse London borough, linked to anonymized electronic mental health records (“CRIS secondary care database”) were extracted on preconception risk factors including BMI, smoking, alcohol, substance misuse, micronutrient deficiencies and physical health diagnoses for women aged 15-40 with an episode of secondary mental health care (January 2008-December 2018) and no pregnancy codes (n=3,633) and a 4:1 age-matched comparison cohort (n=14,532) .

**Results:**

Women in contact with mental health services (whether with or without SMI diagnoses) had a higher prevalence of all risk factors and physical health diagnoses studied after adjustment for deprivation and ethnicity. Women from minority ethnic groups [79.5% of total sample] were less likely to be diagnosed with depression in primary care compared to White British women [adj OR 0.66 (0.55- 0.79) p<0.001] and Black women were more likely to have a severe mental illness [adj OR 3.41(2.63-4.43), p<0.001]. Black and Asian women were less likely to smoke or misuse substances and more likely to be vitaminD deficient. Black women were also significantly more likely to be overweight [adj OR 4.56(3.96-5.24 p <0.001] and have two or more physical health conditions [adj OR 2.98(2.19-4.07) p<0.001] than White British women after adjustment for deprivation and SMI diagnoses.

**Conclusions:**

Our results highlight a need for culturally centered integrative models of care across primary and secondary mental health services.

**Disclosure:**

Closing the Gap is funded by UK Research and Innovation and their support is gratefully acknowledged (Grant reference: ES/S004459/1). Any views expressed here are those of the project investigators and do not necessarily represent the views of the Closing

